# Homoeolog-specific transcriptional bias in allopolyploid wheat

**DOI:** 10.1186/1471-2164-11-505

**Published:** 2010-09-17

**Authors:** Alina R Akhunova, Rustam T Matniyazov, Hanquan Liang, Eduard D Akhunov

**Affiliations:** 1 Integrated Genomics Facility, Throckmorton Plant Sciences Center, Kansas State University, Manhattan, KS 66506, USA; 2 Department of Plant Pathology, Throckmorton Plant Sciences Center, Kansas State University, Manhattan, KS 66506, USA

## Abstract

**Background:**

Interaction between parental genomes is accompanied by global changes in gene expression which, eventually, contributes to growth vigor and the broader phenotypic diversity of allopolyploid species. In order to gain a better understanding of the effects of allopolyploidization on the regulation of diverged gene networks, we performed a genome-wide analysis of homoeolog-specific gene expression in re-synthesized allohexaploid wheat created by the hybridization of a tetraploid derivative of hexaploid wheat with the diploid ancestor of the wheat *D *genome *Ae. tauschii*.

**Results:**

Affymetrix wheat genome arrays were used for both the discovery of divergent homoeolog-specific mutations and analysis of homoeolog-specific gene expression in re-synthesized allohexaploid wheat. More than 34,000 detectable parent-specific features (PSF) distributed across the wheat genome were used to assess *AB *genome (could not differentiate A and B genome contributions) and *D *genome parental expression in the allopolyploid transcriptome. In re-synthesized polyploid 81% of PSFs detected mid-parent levels of gene expression, and only 19% of PSFs showed the evidence of non-additive expression. Non-additive expression in both *AB *and *D *genomes was strongly biased toward up-regulation of parental type of gene expression with only 6% and 11% of genes, respectively, being down-regulated. Of all the non-additive gene expression, 84% can be explained by differences in the parental genotypes used to make the allopolyploid. Homoeolog-specific co-regulation of several functional gene categories was found, particularly genes involved in photosynthesis and protein biosynthesis in wheat.

**Conclusions:**

Here, we have demonstrated that the establishment of interactions between the diverged regulatory networks in allopolyploids is accompanied by massive homoeolog-specific up- and down-regulation of gene expression. This study provides insights into interactions between homoeologous genomes and their role in growth vigor, development, and fertility of allopolyploid species.

## Background

Genetic redundancy created by allopolyploidy is a source of new variation as well as the molecular basis for functional evolution [1-3]. The evidence for several rounds of recent and ancient polyploidization events found by analyzing the genomic sequence data suggests the importance of whole-genome duplication in the evolutionary success of angiosperms [4,5]. Analysis of natural and re-synthesized allopolyploids demonstrated that the combination of homoeologous genomes results in "genomic shock" accompanied by structural rearrangements [6,7], activation of transposons [8], expression changes [7,9,10] and epigenetic modifications [11-13]. Such changes are suggested to lead to the functional diversification of duplicated genes thereby promoting the establishment of new regulatory interactions and, ultimately, are responsible for phenotypic variability and the broader adaptability of allopolyploid species [5]. At a physiological level, allopolyploidy is often associated with plant vigor, adaptation to a broad range of environmental stress factors, resistance to pathogens, and increased fecundity and fertility [1,2,5].

While our knowledge of the molecular basis of phenotypic effects observed in allopolyploids is limited, *cis*- and *trans*-regulatory divergence between parental genomes and their interaction were shown to be important for transcription regulation in inter-species hybrids [14,15] and allopolyploids [16]. The role of *cis*-regulatory divergence between homoeologous genes in polyploids is demonstrated by transcriptional subfunctionalization and neofunctionalization of homoeologs in polyploid cotton [16], tissue- and development-dependent regulation of homoelogous genes in wheat [13,17] and *Brassica *[18]. Regulatory modifications can be facilitated by epigenetic mechanisms [12,13] that were also shown to be involved in regulation of circadian rhythms and growth vigor in *Arabidopsis *hybrids and allopolyploids [11]. Additionally, expression divergence between parental lines has correlated with non-additive gene expression in inter-species hybrids [15] and allopolyploids [9]. Recent studies in cotton using homoeolog-specific gene expression assays highlighted the importance of homoelogous genes in plant development and evolution of the allopolyploid transcriptome [10,19].

The genomic, genetic and cytogenetic resources developed for wheat and its wild ancestors make them an excellent system to study the mechanisms of allopolyploidy-induced processes. Evolutionary history of hexaploid wheat includes two polyploidizations events. The first event occurred about 0.5-0.36 million years ago (MYA) [20,21] involving hybridization of two diploid species *T. urartu *(*A *genome diploid ancestor, genome formula *A*^u^*A*^u^) and *Ae. speltoides *(closely related to the diploid ancestor of the wheat *B *genome, genome formula *SS*) resulting in origin of wild tetraploid wheat *T. dicoccoides *(wild emmer, genome formula *BBAA*) [22,23]. Hexaploid *T. aestivum *originated by the hybridization of cultivated tetraploid wheat *T. turgidum *(domesticated emmer, genome formula *BBAA*) with diploid goatgrass *Ae. tauschii *about 8,000 years ago [24]. In spite of significant reduction of genetic diversity during domestication bottleneck [25] polyploid wheat demonstrates broad range of phenotypic variability and ability to adapt to diverse environmental conditions [26] attributed to the plasticity of the allopolyploid genome. Analysis of gene expression in wheat allopolyploid showed that 16% of genes are expressed non-additively compared to the parental lines [27]. The cDNA-AFLP analysis found suppression of 11.4% and 3.3% of bands in the *D *and *AB *genomes, respectively, and induction of only 0.4% of bands in the *AB *genomes of the allopolyploid wheat [28]. When more distant diploid relatives of wheat genomes *Ae. sharonensis *(genome formula *S*^l^*S*^l^) and *T. monococcum *(genome formula *A*^m^*A*^m^) were used to synthesize allotetraploid wheat the alteration of gene expression was observed for 60 out 3072 (2%) AFLP bands [6]. Recently, Affymetix arrays were used to assess the expression in allopolyploid wheat [29] to show that only 7% of genes show non-additive type of gene expression. Although these studies demonstrate the impact of genome doubling on gene regulation in allopolyploid wheat, there are currently no large-scale studies focusing on genome-wide assessment of genome-specific expression in allopolyploid wheat.

Homoeologous gene expression in polyploids can be assessed using various SNP-based assays [16], expression and genotyping arrays [30,31] or next-generation sequencing technologies [32,33]. Recently, microarrays interrogating homoeolog-specific mutations were successfully used for estimating the relative contribution of homoeologous genes to the cotton transcriptome [10,19]. However, most of these assays require prior information about the homoeolog-specific mutations, which is currently limited for the wheat genome. The Affymetix microarrays have also been successfully used for the discovery of single feature polymorphisms (SFP) and genotyping crop species with large genomes for which, at the time when studies were performed, no complete genome sequences were available [34-38]. Here we applied Affymetrix expression array platform to discover homoeolog-specific mutations and use them to assess the relative expression of homoeologous gene copies in synthetic polyploid wheat created by the hybridization of Tetra-Cantach, the tetraploid derivative of hexaploid wheat, and the diploid ancestor of the wheat *D *genome *Ae. tauschii *[39]. Compared to extracted tetraploid parent the synthetic line showed normal growth vigor and fertility [39] and, therefore, is an excellent system to the study the role of homoeologous gene expression in physiological and morphological changes associated with polyploidization.

## Methods

### Plant material

Microarray hybridization experiments were conducted with the diploid ancestor of the wheat *D *genome *Ae. tauschii *(2n = 14, genome formula *DD*, accession TQ20), reconstituted tetraploid wheat Tetra-Cantach (2n = 28, genome formula *BBAA*) representing the extracted tetraploid component of the hexaploid wheat genome and synthetic hexaploid wheat (2n = 42, genome formula *BBAADD*) created by hybridization of Tetra-Cantach with TQ20 accession of *Ae. tauschii *[39]. This exact set of parental lines and the derived synthetic wheat have been previously characterized morphologically [39], cytogenetically and genetically [40].

### Microarray hybridization

Affymetrix GeneChip Wheat Genome Arrays containing 61,178 probesets were hybridized with RNA isolated from the leaves of 4 week-old seedlings of *Ae. tauschii*, Tetra-Cantach and synthetic hexaploid wheat (henceforth referred to as *AT*, *TC *and *SN*). The plant leaves were collected at the four-leaf stage at the same time-point to avoid the effect of expression fluctuation during the day. Since at this stage wheat produces leaves with the rate of about one leaf every 4-5 days to total of 8-9 leaves before transition to tillering, our sampling point corresponds to the middle of the leaf development stage. This sampling approach should minimize the developmental effects on expression differences among lines. Additionally, this sampling time-point allows us to compare our results with the previously published studies with similar sampling time-points [27]. Four independent biological replicates for each line and 1:1 mixture of *AT *and *TC *RNA were used in the experiment. The fresh plant leaves were flash-frozen in liquid nitrogen and ground with mortar and pestle to obtain a fine powder. RNA was isolated from 100 mg of ground tissue using QIAGEN RNAesy Plant Mini Kit (Qiagen) according to the manufacturer recommended protocol. The intactness of RNA samples was assessed using the Agilent Bioanalyzer 2100 (Agilent Technologies). RNA labeling and hybridization were performed according to standard Affymetrix protocols.

### Data analyses

The Affymetrix arrays were used to obtain genome wide estimates of gene expression in a synthetic wheat line and its parents. Microarray intensity *Cel *files were background corrected and quantile normalized using the rma2 procedure implemented in the R package *affy *[41,42]. The comparison of expression between the synthetic wheat and its parents was performed using only perfect match (PM) probes (61,178 probesets × 11 probes = 672,958 array features). For all PM probes we fit the linear model Y*_ijn _*= μ + *p_i _*+ *g_j _*+ ε*_ijn_*, where Y*_ijn _*is the transcript abundance in replicate *n *(*n *= 1, ..., 4), μ is the overall mean of probe intensity, *p *is the effect of probe *i *(*i *= 1, ..., 11), *g *is the effect of genotype *j*, and ε is the error. The method of contrasts was used to compare the effect of genotype in *AT *vs. *TC *and *SN *vs. *AT *+*TC *comparisons. The false discovery rate was controlled using the Benjamini & Hochberg method maintaining FDR at level 0.05 [43].

**Figure 1:**
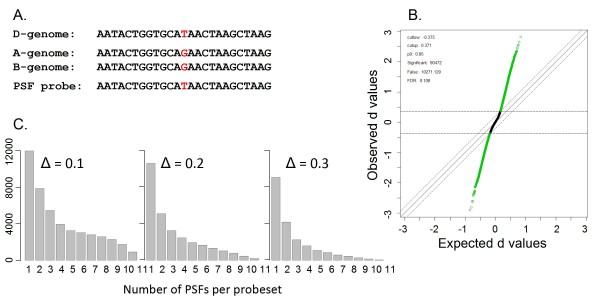
**Identification of parent-specific features (PSFs) in diploid *Ae. tauschii *and tetraploid wheat Tetra-Cantach**. **A**. Alignment of an *AT*-specific probe with the target sequences in the D-genome of *Ae. tauschii *and AB-genomes of Tetra-Cantach. Homoeolog-specific mutation discriminating both the A- and B-genomes from the D-genome is shown in red. **B**. The scatter plot of observed versus expected *d*-statistics obtained for each PM probe. Expected values were obtained by permutations of dataset. Threshold Δ = 0.2 was applied for identifying significantly different oligonucleotide features between *Ae. tauschii *and Tetra-Cantach. **C**. Frequency distribution of the number of PSFs per probeset under various values of threshold Δ.

The Affymetrix data obtained for *AT *and *TC *RNA samples was used to identify oligonucleotide features hybridizing with sequences carrying mutations differentiating the *AT *(*D *genome) and *TC *(*AB *genomes) (Fig. 1A). The statistical procedure for identification of these mutations was similar to that used for identification of single-feature polymorphisms (SFPs) [37,44]. However, the term "SFP", which refers to an oligonucleotide feature differentially hybridizing with different genotypes due to intra-species polymorphisms, in its original meaning is not strictly applicable to our experiment. Most of the mutations differentiating *AT *and *TC *parents are fixed divergent mutations accumulated after the radiation of the diploid ancestors of the wheat *A*, *B *and *D *genomes. Therefore, the probes differentially hybridizing with *AT *and *TC *will be referred to as parent-specific features (PSF) or parent-specific probes. For all 672,958 PM probes on the array, we fit the same linear model Y*_ijn _*= μ + *p_i _*+ *g_j _*+ ε*_ijn _*described above. Residuals containing the probe by genotype effect were tested for difference between genotypes by calculating the *d*-statistics [45] using the Significance Analysis of Microarrays (SAM) procedure implemented in the R package *siggenes *(Fig. 1B). The threshold Δ = 0.2 was selected to identify significantly different features with 0.1 false discovery rate (FDR). Parent-specific oligonucleotide features having higher hybridization intensity with *Ae. tauschii *had negative values of *d*-statistics (henceforth, *AT*-specific probes); PSFs showing higher hybridization intensity with Tetra-Cantach had positive values of *d*-statistics (henceforth, *TC*-specific probes). It is worth mentioning that the *TC*-specific probes cannot discriminate the level of expression of homoeologous genes in the *A *and *B *genomes of tetraploid *TC*. The intensity of *TC*-specific probes on the array is a product of hybridization of transcripts generated by either one of the genomes of tetraploid *TC *or by both of its genomes. The *TC*-specific probes can have greater hybridization intensity with targets in *TC *than with those in *AT *due to either 1) the perfect match of a *TC*-specific probe with both homoeologous sequences in the *A *and *B *genomes and presence of a mismatch in the *D *genome or 2) the perfect match of a probe with either of the two homoeologous sequences in the *A *and *B *genomes and presence of a mismatch in the *D *genome. Likewise, the *AT*-specific probes on the array hybridize more efficiently with *AT *due to the perfect match of a probe with *AT *transcripts and the presence of mismatch in either one or both *TC *genomes. The example of a *AT*-specific probe sequence perfectly matching the *AT *sequence and mismatching the *A *and *B *genome sequences in *TC *is depicted in the Fig. 1A.

The PSFs were experimentally verified by comparing the sequences of 25-mer PM probes with the cDNA sequences of *Ae. tauschii *(*D *genome ancestor) and the two lines of hexaploid wheat *Triticum aestivum *cv. Chinese Spring and *T. aestivum *cv. Jagger. The sequencing of full-length normalized cDNA libraries was performed using the pyrosequencing instrument GS LR70 (Roche) in the University of Oklahoma following the standard single read shotgun 454 sequencing protocol with LR70 kit (Roche). Plants were grown in greenhouse conditions under natural day light supplemented with artificial source of light. The total RNA was isolated from 4 week seedlings using the RNeasy Plant Mini Kit (QIAGEN). First-strand cDNA synthesis was performed according to SMART cDNA synthesis technology (Clontech Laboratories, Inc.) using modified 3' SMART CDS Primer II A (5'-AAGCAGTGGTATCAACGCAGAGTACTTTTGT(9)C T(10)VN-3') and SuperScript III reverse transcriptase (Invitrogen). Double-stranded cDNA was amplified by long-distance (LD) PCR using Advantage 2 PCR Enzyme System (Clontech Laboratories, Inc). Amplification was performed on thermal cycler (Applied Biosystem) with the following PCR parameters: 95°C for 1 min. followed by 16 cycles of 95°C - 15 sec., 65°C - 30 sec., 68°C - 6 min. The quality of double-stranded cDNA was checked on the 1.1% agarose/EtBr gel in 1× TAE buffer and purified with QIAquick PCR Purification Kit (QIAGEN).

The double-stranded cDNA was normalized using TRIMMER cDNA normalization Kit (EVROGEN), which is based on a unique DSN (duplex-specific nuclease) normalization technology and is specifically developed for normalization of cDNA enriched with full-length sequences. The program Lucy 1.19p was used for quality trimming of 454 sequence reads prior to further analyses. PM probe sequences were downloaded from the Affymetrix website [46] and compared with 454 sequence reads using the blastn program with e-value threshold set at 10^-4^. This e-value threshold was selected to allow for no more than one mismatch per 25 bp alignment.

In order to measure the expression of homoeologous genes in the synthetic polyploid we used an approach similar to that applied for assessing the allele-specific expression in a cross of two unrelated strains of *Saccharomyces cerevisae *[30]. Ronald et al (2005) used the observed to expected intensity of probe hybridization to assess the contribution of parental alleles to overall gene expression. The expected intensity of probe hybridization was modeled by taking into account the energy of RNA-DNA duplex formation during hybridization. This model considers two modes of probe hybridization, gene-specific binding and non-specific binding. The former refers to RNA-DNA duplex formation with complementary sequences and the latter refers to the duplex formation with many mismatches. The model ignores the duplexes with few mismatches assuming that probes are preselected against this type of binding [47]. However, in allopolyploid wheat, the presence of recently diverged homoeologous copies of genes makes this model's assumption invalid.

Therefore, to measure the expression of a homoeologous gene relative to total gene expression we used the following approach. All eleven PM probes within each probeset were classified into two categories based on difference in hybridization intensity between parental lines (using previously described *d*-statistics): those that showed detectable differences were called PSFs and those that did not show detectable differences were called non-PSFs. The averaged hybridization signal of non-PSFs (*Ē_t_*) was used to get an estimate of the total gene expression for each probeset. The contribution of *AT *or *TC *copies of a gene to total gene expression was assessed from the ratio of the PSF intensity *E_p _*to the averaged non-PSF intensity *Ē_t _*(henceforth *E_p_/Ē_t _*ratio). The ratio was calculated using the formula *E_p_/Ē_t _*= log_2_*E_iks _*- log_2_*Ē_ims_*, where *E_iks _*is the hybridization intensity of the parent-specific probe *k *in the probeset *i *(i = 1, 2, ..., 21,379) for species *s *(*s *= *AT*, *TC *or *SN*) and *Ē_ims _*is the mean intensity of remaining *m *probes in the probeset *i *in species *s*. The linear model Y*_jn _*= μ + *g_j _*+ ε*_jin _*was fit to *E_p_/Ē_t _*ratio data, where Y*_jn _*is the *E_p_/Ē_t _*ratio in replicate *n *(*n *= 1, ..., 4), μ is the overall mean of *E_p_/Ē_t _*ratio, *g *is the effect of genotype *j *(*j *= *AT*, *TC *or *SN*), and ε is the error. Contrasts were used to compare the intensity ratios in *AT *vs. *SN*, *TC *vs. *SN*, *SN *vs. (*AT *+ *TC)/*2 and *SN *vs. (1/3*AT *+2/3*TC*) comparisons. The latter two contrasts were used to test the different ratios of parental gene expression in a synthetic polyploid. According to previous studies of allopolyploids, including wheat [9,27], 1 : 1 mid-parent model is the best fit to the expression values observed in allopolyploids. The false discovery rate (FDR) among significant features was estimated separately for each contrast using the Benjamini & Hochberg method maintaining FDR at level 0.05 [43]. The ability of *AT*- and *TC*-specific probes to correctly predict the expression divergence from the mid-parent value was verified experimentally by hybridizing Affymetrix microarray chips with the 1:1 mix of *AT *and *TC *RNA samples and fitting the model assuming the 1:1 ratio of *AT *and *TC *expression in the mix. The proportion of significant oligonucleotide features in this experiment to the proportion of significant oligonucleotide features in the contrast *SN *vs. (*AT *+ *TC)/2 *corresponds to the experimentally determined false positive rate.

One of the limitations of the approach used in our study is that the *E_p_/Ē_t _*ratio does not discriminate the expression of the *A *genome homoeolog from that of the *B *genome. The hybridization intensity signal of *TC*-specific probes is composed of two components: one is contributed by hybridization with the *A *genome transcripts and another is contributed by the hybridization with the *B *genome transcripts. Another limitation of the wheat Affymetrix microarray platform for the analysis of homoeologous gene expression is that each identified PSF is efficient for measuring the expression of gene copies from only one of the parental lines, *AT *or *TC*, depending on which one of them have sequences with a perfect match to the PSF. The absence of a probe that matches the sequences of another parent precludes the estimation of expression of both parental gene variants in the polyploid. Therefore, we used *AT*-specific probes for assessing only *AT*-type expression (or *D *genome expression) and *TC*-specific probes for assessing only *TC*-type expression (combined expression of both *A *and *B *genomes) in the synthetic allopolyploid wheat.

### Distribution of parent-specific features across the wheat genome

The blasn program was used to compare the sequences of ESTs used for the Affymetrix probe design with the sequences of ESTs mapped to the wheat deletion bin map [48]. The best blast hits passing the e-value threshold set at 10^-10 ^and having the alignment length more than 100 bp were selected. The bin map locations were extracted from the GrainGenes database [49]. Only those loci that mapped to the deletion bins in synthenic positions on the same homoeologous chromosome were retained for further analysis. Bins were grouped according to the distances of their midpoints from the centromere into five equal intervals along chromosome arms. The number of mapped ESTs matching the Affymetrix probesets was calculated in each of five intervals.

### Quantitative RT-PCR

We performed a two-step RT-PCR in which RNA was first converted into cDNA and then amplified with homoeolog-specific primers. The reaction was performed on the BioRad iQ iCycler real time PCR system. The transcript levels were determined in *SN *and 1:1 mixture of *AT *and *TC *RNA using the SYBR Green detection system. The primers for amplification were designed to harbor homoeolog-specific mutations at their 3'-ends. The mutations differentiating homoeologous sequences were identified by comparing EST sequences generated by 454 sequencing of transcriptomes of hexaploid wheat and its diploid ancestors. The ability of the primers to specifically amplify targets in the parental genomes was tested by PCR with *AT *and *TC *cDNAs. The primer amplification efficiency (*E*) was tested on series of 2-fold diluted cDNAs.

For accurate relative quantification of expression levels, we calculated the theoretical value R_0 _using the formula R_0 _= R_Ct _(1 + *E*)^-Ct^, where R_0 _is the starting fluorescence, R_Ct _is the fluorescence at the threshold cycle Ct and *E *is the amplification efficiency [50]. This method of quantification takes into account the effect of amplification efficiency on the accumulation of PCR products. All R_0 _values were normalized relative to the R_0 _obtained for the actin gene followed by log-transformation. The log-transformed R_0 _values obtained for *SN *were compared with R_0 _values obtained for 1:1 mixture of *AT *and *TC *RNAs. The difference in expression levels was tested using the one-tailed t-test statistics assuming equal variance.

### Functional annotation

Affymetrix probesets containing parent-specific probes were classified into functional categories using the TIGR Gene Ontology assignments [51]. The contigs of wheat genes used for the array design were downloaded from the Affymetrix website and compared with the sequences of TIGR tentative contigs using the blastn program. The blast hits were filtered using the *e*-value threshold 10^-10 ^and an alignment length above 80 bp. The tentative contigs showing the best blast hits were then selected to extract the TIGR GO assignments. The gene annotations were mapped to high-level broader parent terms, referred to as GO Slim terms, using the GO Slimmer tool [52]. Additional functional annotations were performed using the MapMan software [53]. MapMan's Scavenger module was used to assign the genes on the Affymetrix chip to non-redundant functional categories. The Wilcoxon rank-sum test was used to identify functional bins that demonstrate the coordinated change in the expression of multiple genes that belong to the same pathway.

## Results

### Discovery and validation of probes differentially hybridizing with the *AT *and *TC*

We used the PM probes on the wheat array for the discovery of probes hybridizing to sequences harboring mutations that differentiate *TC *(*AB *genome) and *AT *(*D *genome) (Fig. 1A). Background corrected, normalized and log2 transformed expression values of PM probes were extracted from each of 61,127 probesets on the Affymetrix wheat array. The data was analyzed using the linear model described in the Materials and Methods. A total of 90,472 PSFs distributed among 28,679 probesets were discovered at 0.1 FDR (Fig. 1B). Most of the probesets (63%) contained two or more PSFs (Fig. 1C).

For further analysis, we selected only 21,379 probesets with no more than 4 PSFs per probeset. This cutoff value is based on the expected number of nucleotide substitutions in a probeset given the nucleotide substitution rate and the divergence time of diploid ancestors of polyploid wheat [20]. The total length of each probeset is 275 bp (11 probes × 25 bp = 275 bp), mostly represented by coding sequences or non-coding 3'-UTRs. If we assume that the neutral mutation rate in non-coding wheat sequences is 5.5 × 10^-9 ^per site per year (the mutation rate in coding regions is significantly lower) and set the divergence time of the diploid ancestors of the wheat genomes to 2.7 million years [20], we would expect no more than 4 mutations within the 275 bp sequence. This procedure should eliminate probesets that contain excessive number of parent-specific features due to their hybridization with the transcripts encoded by the diverged copies of multigene families. Also, by excluding probesets with more than 4 PSFs we eliminate probes that were falsely detected as PSFs if a gene was silenced or expressed at the low level in one parent, but was expressed at high level in another parent. Under this scenario, all probes in a probeset would appear as PSFs. The resulting dataset consisted of 40,281 PSFs including 18,293 *AT*-specific features and 21,988 *TC*-specific features (Additional File 1).

This dataset was used for experimental validation of discovered parent-specific features by comparing oligonucleotide feature sequences with the Expressed Sequence Tags (ESTs) generated using the GS LR70 pyrosequencing platform (also referred to as 454). A total of 270,230 raw reads were generated with an average length of 216.3 nucleotides (Table 1). After quality filtering to remove adaptors, polyA tails, and low quality sequences, we obtained 224,926 reads with the average length 211 bp totaling to 47,699,354 bases (Table 1). A total of 10.1, 20.6 and 17 Mb of cDNA sequence data was obtained for the diploid ancestor of the wheat *D *genome *Ae. tauschii *and the two lines of hexaploid wheat *T. aestivum *cv. Chinese Spring and *T. aestivum *cv. Jagger, respectively. The sequencing data have been submitted to the NCBI Short Read Archive (accession number SRA012746).

**Table 1:** 454 transcriptome sequencing of *Ae. tauschii *and wheat cultivars Chinese Spring and Jagger

Summary of 454 run	AT	CS	JG	Total
No. raw reads	61,664	113,594	94,972	270,230
Total length of raw reads, bp	12,931,816	24,692,552	20,878,927	58,503,295
No. trimmed reads	48,126	97,148	79,652	224,926
Total length of trimmed reads, bp	10,112,016	20,553,679	17,033,659	47,699,354
Average length of trimmed reads, bp	210	211	214	212
Median length of trimmed reads, bp	211	214	216	214

The sequences of 40,281 PSFs were compared with the 454 sequence reads using the blastn program applying e-value threshold 10^-4^. Eighty one PSFs producing significant 25 bp alignments with the cDNAs of *Ae. tauschii *and both wheat lines were classified into the two groups (Additional File 2). The first group included 39 PSFs that are expected to be *TC*-specific. These PSFs had one mismatch with a *Ae. tauschii *cDNA sequence and at least one mismatch and one perfect match with cDNA sequences of both wheat lines. The second group included 42 PSFs (expected to be *AT*-specific) that have a perfect match with *Ae. tauschii *cDNA sequences and at least one mismatch and one perfect match with cDNA sequences of both wheat lines. For all 81 features we compared the value of *d*-statistics (see Material and Methods) and their assignments to the first and second groups (Additional File 2). In the case of perfect concordance between the PSFs identified on the basis of *d*-statistics and DNA sequence data, all probes in the first group should have positive *d *values and the probes in the second group should have negative *d *values. In the first group, 33 out of 39 probes had positive *d *values leaving 6 with negative *d *values; in the second group, 34 out of the total 42 probes had negative *d *values while 8 probes had positive *d *values. Thus, 10% FDR applied in SAM results in 20% (8/(33+8) × 100% ≈ 20%) of falsely-discovered *TC*-specific features and 15.0% (6/(34+6) × 100% = 15%) of falsely-discovered *AT*-specific features.

### Distribution of PSFs across the wheat genome

**Figure 2:**
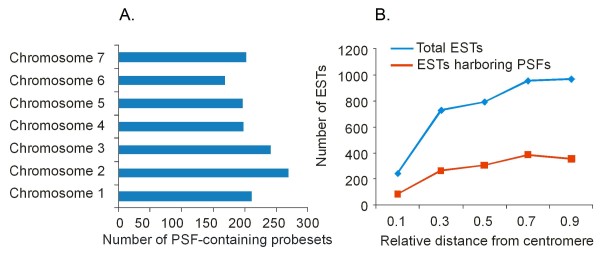
**Distribution of parent-specific features across the wheat chromosomes**. **A**. Distribution of PSF-containing probesets among the 7 homoeologous groups of chromosomes. **B. **Correlation between the distribution of ESTs harboring PSFs and total ESTs along the wheat chromosomes. The distance from the centromere is given in fractions of chromosome arm length where 0 represents centromere and 1.0 represents telomere.

The distribution of the discovered 40,281 PSFs across the wheat genome was inferred by comparing the EST sequences used for Affymetrix array design with the sequences of deletion-bin mapped ESTs [49]. A total of 1,906 PSF-containing probesets showed similarity with the mapped wheat ESTs. We excluded those ESTs that were mapped to more than one chromosomal location in the wheat genome and presumably belong to multigene families. The remaining 1,401 ESTs were distributed similarly among all seven homoeologous groups of chromosomes (Additional File 3, Fig. 2A). The distribution of ESTs showing similarity to PSF-containing probesets along the chromosomal arms mirrors the distribution of all single-copy ESTs, with higher number of parent-specific features mapped to the distal 30% of the chromosome arms (Fig. 2B).

### Using parent-specific features for assessing the homoeolog-specific gene expression

**Figure 3:**
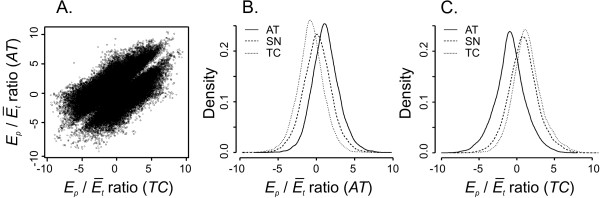
**Comparison of *E_p_/Ē_t _*ratios in *AT*, *TC *and *SN *obtained for 40,281 probes**. **A. **A scatterplot showing the comparison of *E_p_/Ē_t _*ratios calculated for *AT *(*y*-axis) and *TC *(*x*-axis) lines. **B**. Density distribution of *E_p_/Ē_t _*ratios in *AT*, *TC *and *SN *lines calculated for probes having perfect match with the *AT *sequences (*AT*-specific). **C**. Density distribution of *E_p_/Ē_t _*ratio in *AT*, *TC *and *SN *lines for probes preferentially hybridizing with the *TC *sequences (*TC*-specific).

**Figure 4:**
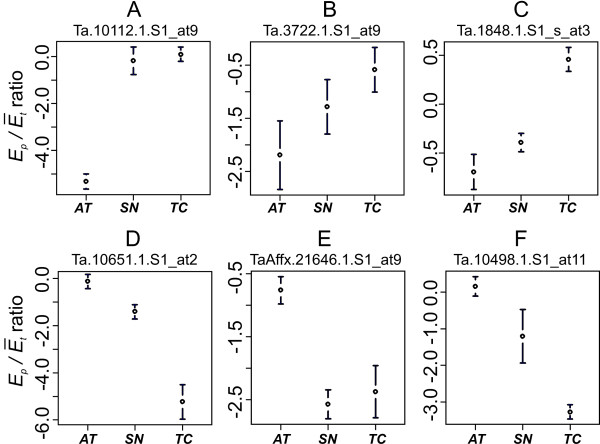
***E_p_/Ē_t _*ratio estimates in *AT*, *SN *and *TC *for selected PSFs**. The graphs are showing the means and 95% confidence intervals of *E_p_/Ē_t _*ratio estimates. The top three probes hybridize preferentially with *TC *(*TC*-specific); the bottom three probes hybridize preferentially with *AT *(*AT*-specific). The names of PSF probes are indicated on the top of each plot.

Given the ability of the Affymetrix microarray to discriminate between *AT- *and *TC- *specific gene expression, we used this platform for measuring the expression of parental genes in the *D *and *AB *genomes of *SN*. The *E_p_/Ē_t _*intensity ratios of PSFs having a perfect match with either *AT *or *TC *target sequences are expected to be higher than the intensity ratios of probes hybridizing with mismatching target sequences (Fig. 3B,C, and 4). This is evident from the well-defined two-cluster pattern produced by plotting the *E_p_/Ē_t _*intensity ratios estimated for 40,281 probes in *AT *(*y*-axis) and *TC *(*x*-axis) lines (Fig. 3A). As expected, the *AT*-specific probes had greater *E_p_/Ē_t _*intensity ratio in *AT *than in *TC *and *TC*-specific probes had greater *E_p_/Ē_t _*ratio in *TC *than in *AT *(Fig. 3A and 4).

The significant fraction of *E_p_/Ē_t _*intensity ratio values in *SN *for *AT*-specific probes falls between the intensity ratios obtained for *AT *and *TC *lines (Fig. 3B), consistent with previous observations that the gene expression in polyploids tends to stabilize at mid-parent level [9,27]. The mean of *E_p_/Ē_t _*intensity ratios in *SN *for *TC*-specific probes was shifted toward *TC E_p_/Ē_t _*intensity ratios (Fig. 3C) suggesting higher level of gene expression in the *A *and *B *genomes combined.

The analysis of contrasts was used to test the two possible ratios of parental gene expression in the synthetic polyploid assuming 1:1 and 1:2 (henceforth, 1:1 and 1:2 models, respectively) *in silico *ratio of *AT *:*TC *gene expression in the *SN *transcriptome. The former model corresponds to cases when parental genomes make equal contribution to the *SN *transcriptiome, whereas the latter model assumes that the tetraploid parent (*TC*) contributes two parts and diploid parent (*AT*) contributes only one part to total gene expression. Based on analysis of contrasts, we classified genes into several expression categories: 1) expression is not significantly different from the assumptions of both 1:1 and 1:2 models (mid-parent expression); 2) expression is significantly different from the assumptions of both models (non-additive expression); 3) expression is not significantly different from the assumptions of 1:1 model but different from 1:2 model; and 4) expression is not significantly different from the assumptions of 1:2 model but different from 1:1 model (Table 2). Both models were fit to the probeset expression data obtained for all genes on the Affymetrix chip and to *E_p_/Ē_t _*ratio data calculated for PSFs (Additional File 4).

**Table 2:** Comparison of the fit of two models to probeset and *E_p_/Ē_t _*ratio expression data

Expression ratio models		1AT : 2TC model			
		**Probeset expression data**		***E***_***p***_***/Ē***_***t ***_**ratio data**	
		
		**Significant**	**Not significant**	**Significant**	**Not significant**

**1AT : 1TC model**	Significant	24,070	8,486	6,430	4,863
	Not significant	2,946	25,676	1,879	21,497

The models assume 1AT : 1TC and 1AT : 2TC expression ratio of parental genes in the synthetic allopolyploid. False discovery rate was controlled at level 0.05.

The analysis of probesets showed that nearly 61% of all genes in *SN *are expressed at mid-parent level corresponding to either 1:1 or 1:2 parental expression ratios. The expression of 13.9% of genes fit the expectations of the 1:2 model but was different from the assumptions of the 1:1 model, whereas 4.8% of genes were expressed at levels that fit the expectations of the 1:1 model but was different from that of the 1:2 model. A total of 39% of genes were expressed at the levels suggesting non-additive mode of expression.

The *E_p_/Ē_t _*ratio dataset was used to assess relative contribution of parent-specific gene expression in allopolyploid wheat transcription. Only 20,643 out of 21,379 previously selected probesets were included into the final analysis (Additional File 4). A total of 736 probesets with more than one PSF per probeset were removed from the analysis because they contained PSFs failing to predict the expression change in *SN *in the same direction relative to the mid-parent value. For example, a probeset that contains two *AT*-specific probes, one predicting the up-regulation of gene expression in *SN *and another down-regulation of gene expression in *SN*, would be removed from dataset. The analysis of *E_p_/Ē_t _*ratio data showed that 81% of homoeologous copies of genes are expressed at the levels fitting the expectations of 1:1 or 1:2 models, and 19% are expressed non-additively. The examples of PSFs detecting the mid-parent levels of homoeologous gene expression are shown in Fig. 4B and 4F. The *AT*-specific probes detected a higher number of genes with mid-parent level of expression in *SN *than the *TC*-specific probes (Table 3). The differences in the proportion of additively and non-additively expressed genes between the entire probeset data and the *E_p_/Ē_t _*ratio data (Table 2) are mostly explained by the filtering step included in the selection of 20,643 PSF-containing probesets for calculation of *E_p_/Ē_t _*ratio. When only these 20,643 probesets were included into the analysis of contrasts, the proportion of genes fitting 1:1 and 1:2 models increased from 61% to 73%.

**Table 3:** Parent-specific gene expression in allopolyploid wheat

Gene regulation	*AT- *type expression	*TC- *type expression
Down-regulated	548 (705)	317 (406)
Up-regulated	1,297 (1,601)	2,988 (3,718)
Mid-parent	11,683 (13,840)	12,388 (14,399)

The table shows the number of homoeologous genes interrogated by PSFs (provided in the brackets) in the *AB *and *D *genomes of *SN*.

The remaining 6,430 PSFs detected the non-additive mode of gene expression in allopolyploid wheat. For *AT*-specific probes, a decrease in the *E_p_/Ē_t _*ratio in *SN *relative to the mid-parent value indicates the decrease of the *AT*-parent contribution to total expression (Fig. 3E), whereas an increase in the *E_p_/Ē_t _*intensity ratio in *SN *relative to the mid-parent value would suggest increase of the *AT*-type part in total gene expression (Fig. 3D). Following the same reasoning, the decrease in the *E_p_/Ē_t _*ratio in *SN *relative to the mid-parent value for *TC*-specific probes is evidence of down-regulation of *TC*-type expression (Fig. 3C), while an increase of *SN E_p_/Ē_t _*ratio relative to the mid-parent value suggests an increase in *TC*-type expression (Fig. 3A). The number of non-additively expressed genes detected by *TC*-specific probes compared to that detected by *AT*-specific probes was almost 2 times higher, which points to differential patterns of homoeologous gene activation and repression in the *D *and *AB *genomes after polyploidization. The proportion of up- and down-regulated probes among non-additively expressed genes was 82.7% and 17.3%, respectively. Out of 2,306 *AT*-specific probes, 705 (30.6%) showed the decrease of *AT*-type expression in *SN*, whereas 1,601 (69.4%) showed the increase of *AT*-type expression in *SN *transcriptome. Among the *TC*-specific probes 4,124, showed statistically significant departure of *E_p_/Ē_t _*intensity ratios from the expected mid-parent values with 3,718 probes (90.2%) showing the increase of *TC *parent contribution to *SN *expression whereas 406 probes (9.8%) demonstrated the decrease of *TC *proportion in total gene expression. The number of genes with increased *TC*-type expression was significantly higher than the number of genes with decreased *TC*-type expression (χ^2^-test, P < 1 × 10^-10^). Out of all up-regulated homoeologous genes, 69.9% showed increased proportion of *AB *genome parental expression and 30.1% showed increased contribution from the *D *genome parent. The opposite trend was discovered for down-regulated genes out of which 63.5% were down-regulated in the *D *genome and 36.5% were down-regulated in the *AB *genomes.

We have experimentally estimated the rate of false positives among the probes capable of detecting the bias from mid-parent gene expression by measuring the expression in the mix of *AT *and *TC *RNA. Four Affymerix microarrays were hybridized with the mix of parental RNA and the *E_p_/Ē_t _*ratios of 34,669 probes were compared between the mix and *in silico *mid-parent values. We fit the linear model that correctly assumes that all genes in the mix are expressed at half the level of gene expression in the parental genomes. The significant probes in this case will correspond to false positives misidentified as cases of parent-specific bias in gene expression. Out of all 34,669 probes 3,804 (~11%) showed intensity ratios that deviate from the expected mid-parent value at FDR 0.05.

We tested the effect of expression divergence between parental genomes on gene expression in the synthetic polyploid wheat. The analysis of Affymetrix data at the probeset level was used to assess the expression divergence between *AT *and *TC *lines. Approximately 57% (34,809/61,178) of genes showed significant difference in the expression level at FDR 0.05. Expression divergence between the parental transcriptomes explains the significant proportion of non-additive expression variation in the synthetic polyploid wheat. Out of 6,430 probes whose *E_p_/Ē_t _*ratio indicates departure from the mid-parent value in *SN*, 83.6% were found in the genes that are also differentially expressed between the *AT *and *TC *parents. Among 1986 PSFs that were located in the genes expressed at higher levels in *AT *than in *TC*, 1,414 and 28 probes were found in the genes that are up- and down-regulated in the *D *genome of *SN*, respectively, and 188 and 356 probes were found in the genes that are up- and down-regulated in the *AB *genome of *SN*, respectively. Among 3,389 PSFs found in the genes expressed at higher levels in *TC *than in *AT*, 32 and 653 PSFs were found in the genes that are up- and down-regulated in the *D *genome of *SN*, while 2,689 and 15 probes were found in the genes that are up- and down-regulated in the *AB *genomes of *SN*, respectively.

We have validated the expression of several parental copies of a random set of genes in *SN *by quantitative RT-PCR assay. A total of 6 genes in the wheat *A*, *B *and *D *genomes representing 4 homoeologous sets of loci were selected and the expression of homoeologous copies of these genes was measured in *SN *and a 1:1 mixture of *AT *and *TC *RNA. Although, previous studies indicate that due to differences in discriminatory power between the RT-PCR and microarray methods, the confirmation rate can vary [54], we were able to confirm expression change for all these genes. In all cases the direction of expression change predicted by microarray hybridization and quantitative RT-PCR were consistent (Additional File 5).

### Functional annotation of homoeologous genes

To better understand how polyploidy impacts the regulation of homoeologous genes involved in various biological processes, we used the GO annotations. In order to map gene annotations to high-level, broader parent terms we used the GO Slimmer tool [52]. The results of this analysis are presented in Table 4 show that the majority of genes subjected to regulatory modifications also belong to most abundant functional categories including genes involved in metabolism, biosynthesis, organism development, cellular organization, translation, transport and stress response. We have tried to identify statistically over- and under-represented functional categories using the Fisher exact test. The *p*-values of the test statistic were corrected for multiple comparisons maintaining FDR at level 0.05. Only one functional category was significantly overrepresented for up-regulated genes (GO:0006091) including *D *genome homoeologous genes involved in the generation of precursor metabolites and energy. The functional category GO:0007049 was over-represented for down-regulated *AB *genome homoeologs involved in cell cycle regulation (Table 4). Among down-regulated genes in the *D *genome of *SN *the genes that are involved in catabolic processes and carbohydrate metabolism were under-represented. Lower level of up-regulation than expected was observed for the D genome homoeologs that belong to translation (GO:0006412) and cell communication (GO:0007154) functional categories. Under-representation of down-regulated *AB *genome homoeologous genes was detected only for amino acid metabolism functional category (GO:0006519). The *AB *genome homoeologs involved in carbohydrate metabolic process (GO:0005975) and cell communication (GO:0007154) were also under-represented among up-regulated genes.

**Table 4:** GO annotation of up- and down-regulated homoeologous copies of genes in the synthetic polyploid wheat

GO term	Biological process	D genome (*AT*)		AB genome (*TC*)	
		
		suppression	activation	suppression	activation
GO:0009987	cellular process	264	512	145	1231
GO:0008152	metabolic process	214	454	137	1039
GO:0009058	biosynthetic process	127	170	53	494
GO:0019538	protein metabolic process	115	199	78	545
GO:0016043	cellular component organization	72	123	39	290
GO:0006412	translation	64	54**	29	232
GO:0006810	transport	61	108	42	287
GO:0006139	nucleic acid metabolic process	55	101	21	255
GO:0006950	response to stress	53	65	24	201
GO:0007275	Multi-cellular organism development	51	101	31	229
GO:0008150	Biological process	45**	118	45	295
GO:0006464	protein modification process	42	95	30	205
GO:0000003	reproduction	38	69	33	154
GO:0009628	response to abiotic stimulus	30	32	15	88
GO:0009790	embryonic development	29	52	24	121
GO:0009791	post-embryonic development	27	41	19	102
GO:0040007	growth	23	50	21	111
GO:0007049	cell cycle	22	49	26*	93
GO:0006091	precursor metabolites and energy	21	62*	4	97
GO:0006350	transcription	19	56	10	106
GO:0006519	cellular amino acid metabolic process	18	17	0**	72
GO:0009653	anatomical structure morphogenesis	17	42	10	78
GO:0007165	signal transduction	17	55	22	139
GO:0009056	catabolic process	14**	98	21	172
GO:0009607	response to biotic stimulus	13	19	6	36
GO:0009719	response to endogenous stimulus	13	24	2	55
GO:0008219	cell death	11	19	7	39
GO:0030154	cell differentiation	11	35	5	71
GO:0016265	death	11	19	7	41
GO:0019748	secondary metabolic process	10	7	0	12
GO:0005975	carbohydrate metabolic process	9**	80	14	94**
GO:0006259	DNA metabolic process	9	17	3	49
GO:0006629	lipid metabolic process	9	24	5	39
GO:0007154	cell communication	8	16**	5	47**
GO:0019725	cellular homeostasis	7	6	4	34
GO:0009605	response to external stimulus	6	10	2	31
GO:0007267	cell-cell signaling	5	4	1	13
GO:0015979	photosynthesis	5	10	3	15
GO:0016049	cell growth	3	10	3	14
GO:0009991	response to extracellular stimulus	3	5	2	17
GO:0007610	behavior	2	9	2	34
GO:0009908	flower development	2	3	0	8
GO:0009838	abscission	1	0	0	1
GO:0040029	regulation of gene expression, epigenetic	1	8	0	11

* GO categories showing statistically significant overrepresentation of genes (Fisher exact test, FDR ≤ 0.05)

** GO categories showing statistically significant underrepresentation of genes (Fisher exact test, FDR ≤ 0.05)

We have also used the Wilcoxon rank-sum test implemented in the MapMan software to identify the sets of co-regulated homoeologous genes assigned to the same functional pathways or genetic networks whose expression deviates from the response of all other genes [55]. The Benjamini and Hochberg correction was used to control for the false discovery rate [43]. The MapMan software performs functional classification of genes using the redundancy-reduced ontology [55]. Contrary to GO annotation, MapMan assigns a gene to the most appropriate functional category (BIN) rather than sorting it into multiple BINs. We decided to test if this reduction of redundancy will improve our ability to identify functional categories of genes that are co-regulated in *SN*. Parent-specific co-regulation of large sets of genes in *SN *was observed for only a few functional categories (Table 5). The coordinated up-regulation of genes involved in protein biosynthesis pathways was observed in the *A *and *B *genomes of *SN*. The genes involved in photosynthetic pathways, protease inhibitor/seed storage/lipid transfer and protein folding processes were also co-activated in the *A *and *B *genomes of *SN*. Contrary to this observation, the *D *genome in *SN *experienced massive down-regulation of genes involved in protein synthesis pathways (Table 5).

**Table 5:** Homoeolog-specific co-regulation of various functional groups of genes in allopolyploid wheat

MapMan BIN	MapMan functional classes	Genome	Expression	P value*
29	Protein biosynthesis	*AB*	up	6.7 × 10^-3^
1.3.1	Photosynthesis: calvin cycle, rubisco large subunit	*AB*	up	8.8 × 10^-3^
1.1	Photosynthesis: light reaction	*AB*	up	0.01
26.21	Protease inhibitor/seed storage/lipid transfer protein (LTP) family protein	*AB*	up	0.02
29.6	Protein folding	*AB*	up	0.05
29.2	Protein synthesis	*D*	down	2.1 × 10^-5^
29.2.1.2	Ribosomal protein synthesis	*D*	down	2.1 × 10^-5^
29.2.1	Protein synthesis, ribosomal proteins	*D*	down	3.2 × 10^-5^
29.2.1.2.2	Ribosomal protein synthesis 60S subunit	*D*	down	0.02

* P value is subjected to Benjamini and Hochberg (1995) correction for multiple test comparison

## Discussion

Most genes in the synthetic allopolyploid wheat were expressed at mid-parent level fitting the expectations of either 1:1 or 1:2 parental expression ratios, which indicates that polyploidization did not have detectable impact on their regulation. A significant amount of functional redundancy retained after polyploidization found in our study was also consistently found in other artificially created polyploids [7,9,27]. This phenomenon apparently underlies the evolutionary flexibility of polyploid wheat [26], its ability to withstand large scale chromosomal deletions or even loss of entire chromosomes [56,57], and to accumulate high density of EMS-induced mutations [58].

The analyses of probeset data resulted in the higher proportion of non-additively expressed genes (39%) in synthetic wheat than that previously detected using long-oligonucleotide microarray platforms in wheat [27,29], Arabidopsis [9] or Brassica [7]. The differences in non-additive gene expression between our study and previously reported estimates can potentially be attributed to technological capacities of short- and long-oligonucleotide microarray platforms and differences between genotypes used to create allopolyploids. The usage of a short oligonucleotide array system sensitive to the presence of mutations discriminating one homoeolog from another may have increased the rate of non-additive expression discovery. In both previous studies [27,29] natural tetraploid wheat accessions were used to create allohexaploid synthetics. Apparently, differences accumulated during the co-evolution of *AB *and *D *genomes of hexaploid wheat contribute to higher proportion of non-additive expression in reconstituted TetraCantach/*Ae. tauschii *allopolyploid observed in our study.

We used an Affymetrix microarray system for the simultaneous detection of divergent nucleotide mutations differentiating the wheat *AB *genomes from the *D *genome and to measure the relative contribution of parents to total expression in the synthetic polyploid wheat. According to the results of quantitative PCR and microarray hybridization with mid-parent mixture of parental RNA samples, this approach can correctly predict significant departure from the expected mid-parent level of expression with 24% false positive rate. The *E_p_/Ē_t _*ratio data was more consistent with the observations made in previous studies; the majority of the parental genes in synthetic polyploid wheat (81%) were expressed at the mid-parent levels corresponding to 1:1 or 1:2 expression ratios between the parental genomes. Non-additive contribution from the *D *or *AB *genome homoeologs was observed for the remaining 19% of genes.

One of the interesting aspects of homoeologous gene expression in allopolyploid species is transcriptional dominance of one of the parental genomes [9,10,19,59]. The bias toward *TC*- type parental expression in allopolyploid wheat suggests the transcriptional dominance of *TC*. Our study demonstrated that out of all genes showing the increase of parental expression in *SN *about 70% were of *TC*-type. In contrast, the *D *genome homoeologs comprised about 63% of all genes showing decreased contribution of parental expression to *SN *transcriptome. Similar proportion of significantly biased genes (80%) was discovered by studying homoelog-specific gene expression in cotton [10]. Several mechanisms have been proposed to account for parent-specific expression bias in allopolyploids including the incompatibility of regulatory elements [28], removal of gene imprinting [17,60,61], chromatin modification [9] and activation of transposable elements adjacent to the genes [8]. Apparently, all of these mechanisms play some role during the adjustment of two diverged parental regulatory systems. Regulation of homoeologous genes in the polyploid wheat suggests that divergent mutations in *cis*- and *trans*-acting regulatory elements or epigenetic modifications can result in spatial and/or temporal partitioning of gene expression [17,60]. Likewise, compensatory co-evolution of *cis*- and *trans*-acting factors in different lineages of Drosophila can lead to dysregulation of about 30% of genes in the hybrids [14]. The role of interaction between the diverged regulatory elements in modulating the expression of homoeologous genes in polyploids is confirmed by our data, which shows that 84% of non-additive changes in gene expression in *SN *can be explained by the transcription divergence between *Ae. tauschii *and Tetra-Cantach. This result is also consistent with the data reported for Arabidopsis and cotton [9,10]. The proportion of genes for which we observed the expression divergence between the parental lines (~57%) was similar to those reported for *Arabidopsis *(47%) and cotton (42-53%) [9,59], and higher than 32% divergence reported for *Brassica *[7]. An even higher level of expression divergence (78%) was reported for *Ae. tauschii *and *T. turgidum *[27] using a long oligonucleotide array system.

Interestingly, transcription divergence has been shown to correlate with the divergence of regulatory elements [62], which, in turn, depends on the evolutionary history of diverged lineages and time passed since their divergence. Recent studies in *Drosophila *demonstrated that the divergence of *cis*-*trans *elements occurs rapidly, resulting in about 40% of genes showing regulatory changes in ~2.5 million years of divergence [14,31]. In genus *Gossypium *(L.), the divergence time between the diploid species (5-10 MYA) was positively correlated with the expression divergence (42% - 53%) [59]. The diploid ancestors of polyploid wheat genomes diverged about 2.7 MYA followed by an initial polyploidization event resulting in the origin of *T. dicoccoides *(*AB *genome) that occurred about 0.5 MYA [20,21]. Therefore, when compared to other inbreeding allopolyploid species, the level of expression divergence reported for tetraploid wheat and the diploid ancestor of the wheat *D *genome shows an acceleration of regulation divergence after the first polyploidization event. The acceleration of expression divergence is probably driven by functional redundancy created by the first round of whole genome duplication and facilitated by epigenetic modifications and regulatory mutations. This process may play important role in gene expression evolution and in the generation of evolutionary novelties and functional variation upon which selection can act.

We hypothesized that compensatory co-evolution of *cis-trans *elements in the parental genomes may lead to the divergence of whole networks of genes that can be regulated independently in allopolyploids. The level of regulatory incompatibility between the networks in allopolyploids probably vary for different gene ontology categories and most likely are defined by the strength of natural selection. Those *cis- *and *trans- *acting elements that experienced strong purifying selection might remain compatible even after a long period of divergent evolution. Experimental studies demonstrated that transcription factors may co-evolve with the subset of targets that they regulate [14,63]. The parent-specific bias in gene regulation documented in allopolyploids can partially be explained by this process [9,19,59]. Additionally, parent-specific regulation of groups of genes could be important for maintaining correct gene dosage. It was hypothesized that stoichiometric ratios in macromolecular complexes or balances of gene products involved in multiple steps in regulatory cascades are targeted by positive selection and important for optimal organism fitness [64,65]. According to the gene balance hypothesis, if the gene product's concentration does not follow stoichiometry of the complex or cascade, then fitness is lowered [65]. One intriguing possibility is that the restoration of fecundity and growth vigor of the extracted tetraploid TetraCantach after hybridization with *Ae. tauschii *is partially the result of gene dosage restoration through adjustment of gene expression.

Remarkably, using the non-redundant functional classification implemented in MapMan software we were able to identify several pathways that showed coordinated changes in gene expression that was restricted to only one of the parental genomes. We observed the coordinated up-regulation of genes involved in photosynthetic and protein biosynthesis and folding pathways in the *A *and *B *genomes of allopolyploid wheat. This process was accompanied by the coordinated down-regulation of protein biosynthesis pathway in the *D *genome. Functional classification using GO categories showed an over-representation of genes involved in the generation of precursor metabolites and energy in the *D *genome of *SN*.

Our ability to identify coordinated regulation probably depends on the relative contribution of *cis*- and *trans*- divergence to gene regulation in allopolyploids. Since mutations in transcription factors have large pleiotropic effects [66], any processes that are regulated by few diverged *trans*- factors that co-evolved with a large set of *cis*-targets should be readily detectable. The prevalence of *cis*-type regulation would localize regulatory effects to the level of a single gene. This type of regulation seems to be a major contributor to gene regulation in allopolyploids [16] and intra- and inter-species hybrids [15,31] and may explain the low number of co-regulated homoeologous genes found in our study. More rigorous testing including various functional classes of genes combined with the analysis of *cis*-regulatory divergence needs to be performed to estimate the relative impact of *cis*- and *trans*-effects on gene regulation in allopolyploids.

Interaction between the diverged ancestral genomes in newly formed allopolyploids plays an important role in evolution by generating new functional variation and contributing to increased vigor and fertility, broader adaptability and phenotypic variability of polyploid species [1,3,67,68]. In yeast, novel *cis*-*trans *interactions established in a hybrid were shown to be involved in environmental response [69]. The plasticity of the wheat genome, arising from genetic and functional variation contributed by allopolyploidy, was considered to be a key factor defining its adaptability to diverse environments [26,70]. One of the mechanisms proposed to explain the growth vigor of *Arabidopsis *allopolyploids and hybrids includes the regulation of photosynthesis and sugar metabolism through the epigenetic modification of circadian clock genes [11]. Similarly, up-regulation of the *AB *genome homoeologous genes involved in protein biosynthesis and photosynthesis may contribute to restoration of growth vigor and fertility in allopolyploid wheat compared to that of tetraploid wheat Tetra-Cantach. Kerber (1964) proposed that the existence of genetic changes in the *AABB *component of the wheat genome accumulated since the origin of hexaploid wheat result in reduced growth vigor and fertility of reconstituted tetraploids which can be compensated by hybridization with *Ae. tauschii*. Our results further suggest that the interaction between the *D *and *AB *genomes is critical in modulating homoeolog-specific expression which is apparently responsible for physiological and morphological characteristics of allopolyploid wheat.

## Conclusions

In summary, the approach was developed that utilizes the Affymetrix array system for measuring homoeolog-specific gene expression in allopolyploid wheat. The expression data showed the dominance of the *AB *genome in the transcriptome of the re-synthesized allopolyploid. The expression divergence between the transcriptomes of parental lines explained most of the observed non-additive expression in allopolyploid wheat. Evidence of homoeolog-specific coordinated up- and down-regulation of several functional gene categories, including those involved in protein synthesis and photosynthesis processes, suggests the co-evolution of *cis*- and *trans*-regulators. Additionally, the co-evolution of *cis*- and *trans*-elements may lead to divergence and incompatibility of regulatory networks in allopolyploids. The up-regulation of protein synthesis and photosynthetic pathways in the *AB *genome of synthetic polyploid wheat that result in an increase in vigor and restoration of fertility provide good targets for studying the molecular basis of these described phenomena in allopolyploids. While this causal relationship still requires experimental verification, it suggests the existence of established regulatory interactions between homoeologous genomes that lead to increased growth vigor, fertility and biomass in allopolyploid wheat.

## List of abbreviations

PSF: parent-specific features; SFP: single-feature polymorphism; MYA: million years ago; PM: perfect match; MM: mismatch

## Authors' contributions

AA prepared samples for Affymetrix hybridization and 454 sequencing, performed validation of Affymetrix results by 454 sequencing, and helped to draft the manuscript; HL performed analysis of 454 sequencing data; RM performed experimental validation of Affymetrix results; EA conceived the experiment, performed the statistical analysis and drafted the manuscript. All authors read and approved the final version of the manuscript.

## Supplementary Material

Additional file 1 **List of 40,281 parent-specific oligonucleotide features**. File contains the list of 40,281 Affymetrix oligonucleotide features differentially hybridizing with *AT *and *TC *parental transcripts. The linear model described in Methods was fit to PM probe intensity data. Residuals containing the probe by genotype effect were tested for difference between genotypes by calculating the *d*-statistics [45] using the Significance Analysis of Microarrays (SAM) procedure implemented in the R package siggenes. The threshold Δ = 0.2 was selected to identify significantly different features at 0.1 false discovery rate. Click here for file

Additional file 2 **Validation of parent-specific features by comparing with 454 sequence data**. Validation of parent-specific features by comparing with 454 sequence data. The table provides the list of PSFs showing significant blast hits with sequence reads obtained for cDNA libraries of *Ae. tauschii*, *T. aestivum *cv. Chinese Spring and *T. aestivum *cv. Jagger; PM - Affymetrix probe perfectly matches the 454 sequence read; MM - Affymetrix probe have a mismatch with the 454 sequence read; d-value is used in SAM for identification of PSFs. Click here for file

Additional file 3 **Distribution of PSFs across the wheat genome**. List of Affymetrix probesets with parent-specific features (PSF), their locations on the deletion bin map and a list of deletion bin mapped ESTs showing similarity to transcripts interrogated by Affymetrix probesets. The map locations were calculated by averaging the deletion bin midpoints of homoeologous chromosomes and assigning them to one of the five intervals (0-0.2, 0.2-0.4, 0.4-0.6, 0.6-0.8, 0.8-1.0). Click here for file

Additional file 4 **Parent-specific gene expression in allopolyploid wheat**. The method of contrasts was used to compare *E_p_/Ē_t _*intensity ratios between allopolyploid wheat and its parents. Two possible ratios of parental gene expression in the synthetic polyploid were tested assuming 1:1 (AT + TC)/2) and 1:2 (1AT+2TC)/3) *in silico *ratio of AT:TC gene expression in the SN transcriptome. The FDR was maintained at 0.05. Click here for file

Additional file 5 **Validation of Affymetrix microarray hybridization results by quantitative RT-PCR**. Expression levels were converted to theoretical value R_0 _using the formula R_0 _= R(Ct) (1 + E)^(-Ct)^, where R_0 _is the starting fluorescence, R(Ct) is the fluorescence at the threshold cycle Ct and E is the amplification efficiency. The R_0 _values were normalized to R_0 _of actin gene followed by log-transformation. The expression levels in SN and 1:1 mixture of AT and TC RNA were compared using the *t*-test. Click here for file
